# Supplementary figures and images for: Breakthrough infection elicits hypermutated IGHV3-53/3-66 public antibodies with broad and potent neutralizing activity against SARS-CoV-2 variants including the emerging EG.5 lineages
Source: PLoS Pathog. 2023 Dec 4;19(12):e1011856. doi: 10.1371/journal.ppat.1011856 (PMC10721163; doi:10.1371/journal.ppat.1011856)

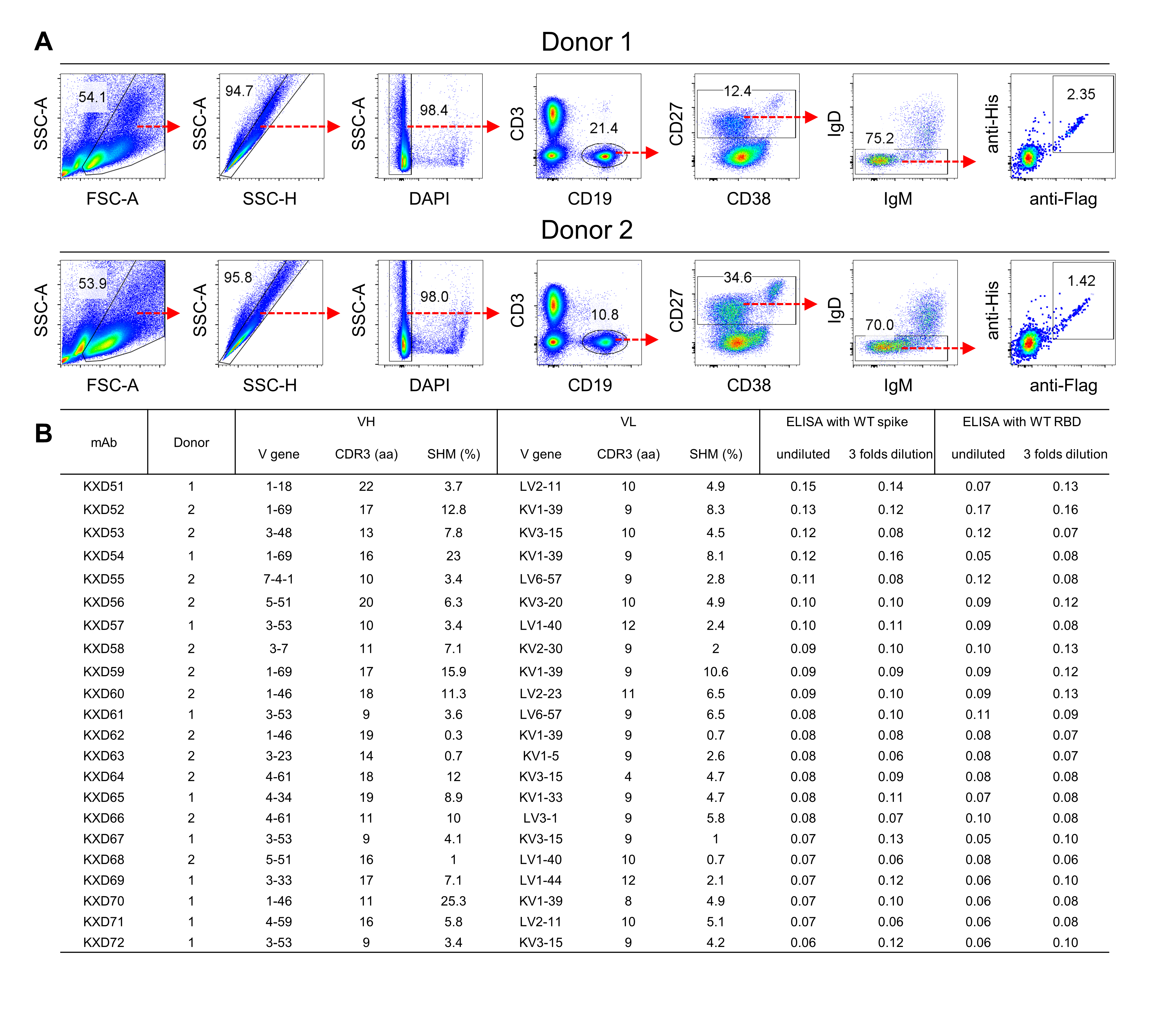

Supplement: S1 Fig — (A) FACS plots representing gating strategies for spike-specific single B cell sorting. (B) Summary of 22 spike-negative mAbs. (TIF) [file ppat.1011856.s001.tif]

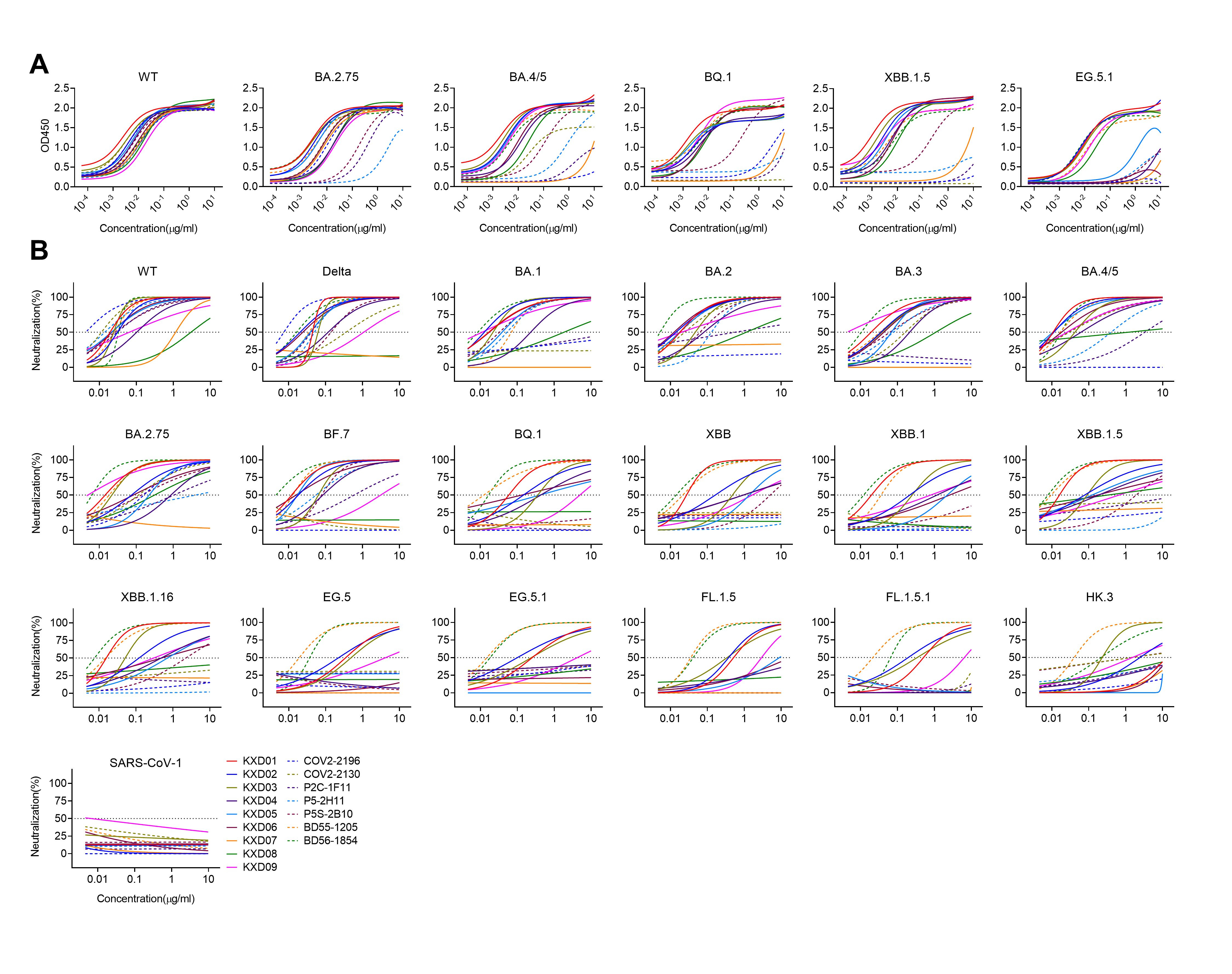

Supplement: S2 Fig — (A) Binding activity against WT and variant RBDs measured by ELISA. (B) Neutralizing activity against a panel of pseudoviruse. The data are represented as non-linear fit curves calculated by least squares fit. All results are representatives of two independent experiments, in which duplicates are performed. (TIF) [file ppat.1011856.s002.tif]

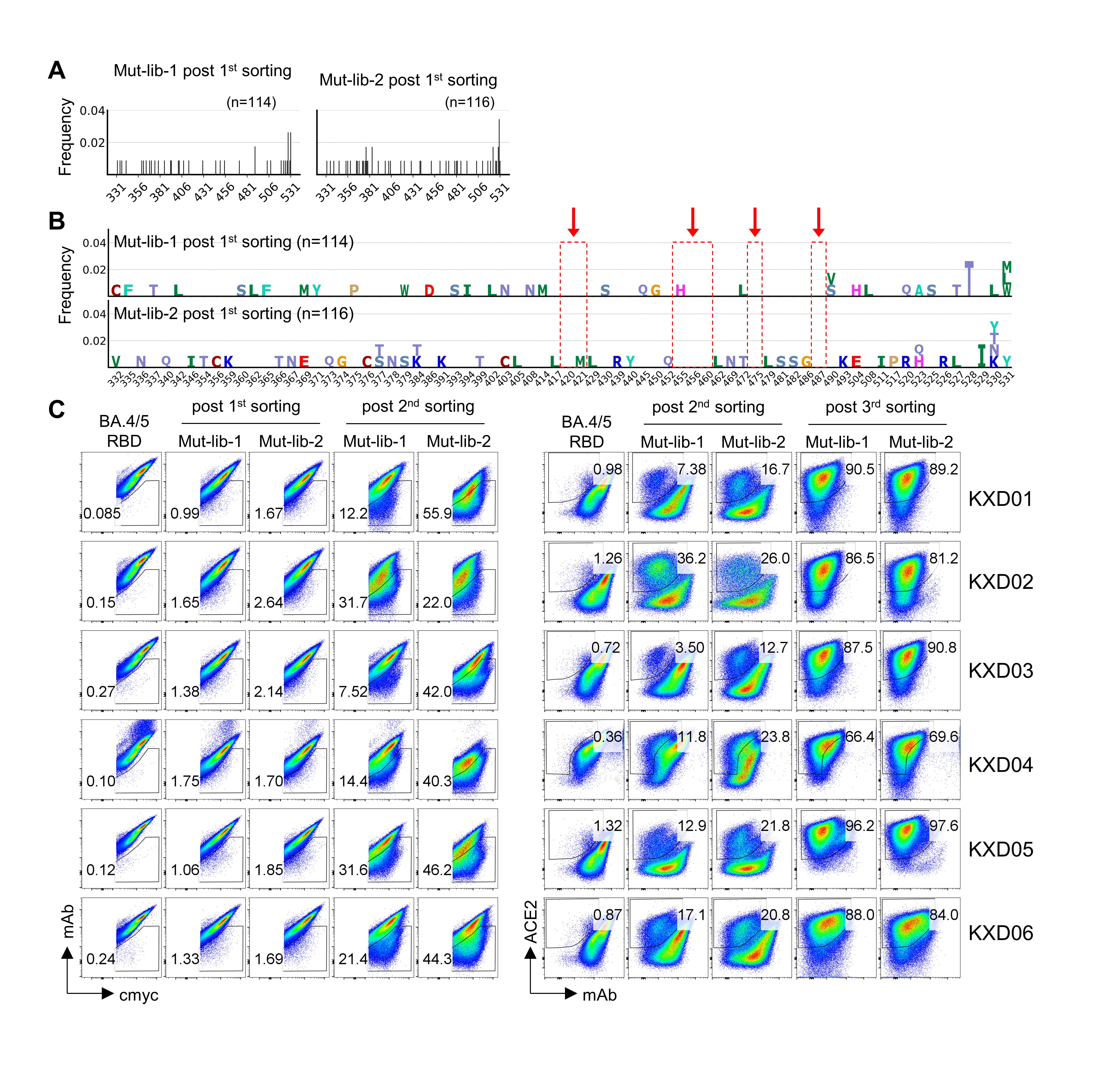

Supplement: S3 Fig — (A) Sequence analysis of mutants post the first sorting. (B) Mutation profile of yeasts post the first sorting. (C) FACS plots of the second and the third sorting of two RBD mutant libraries by KXD01-06. (TIF) [file ppat.1011856.s003.tif]

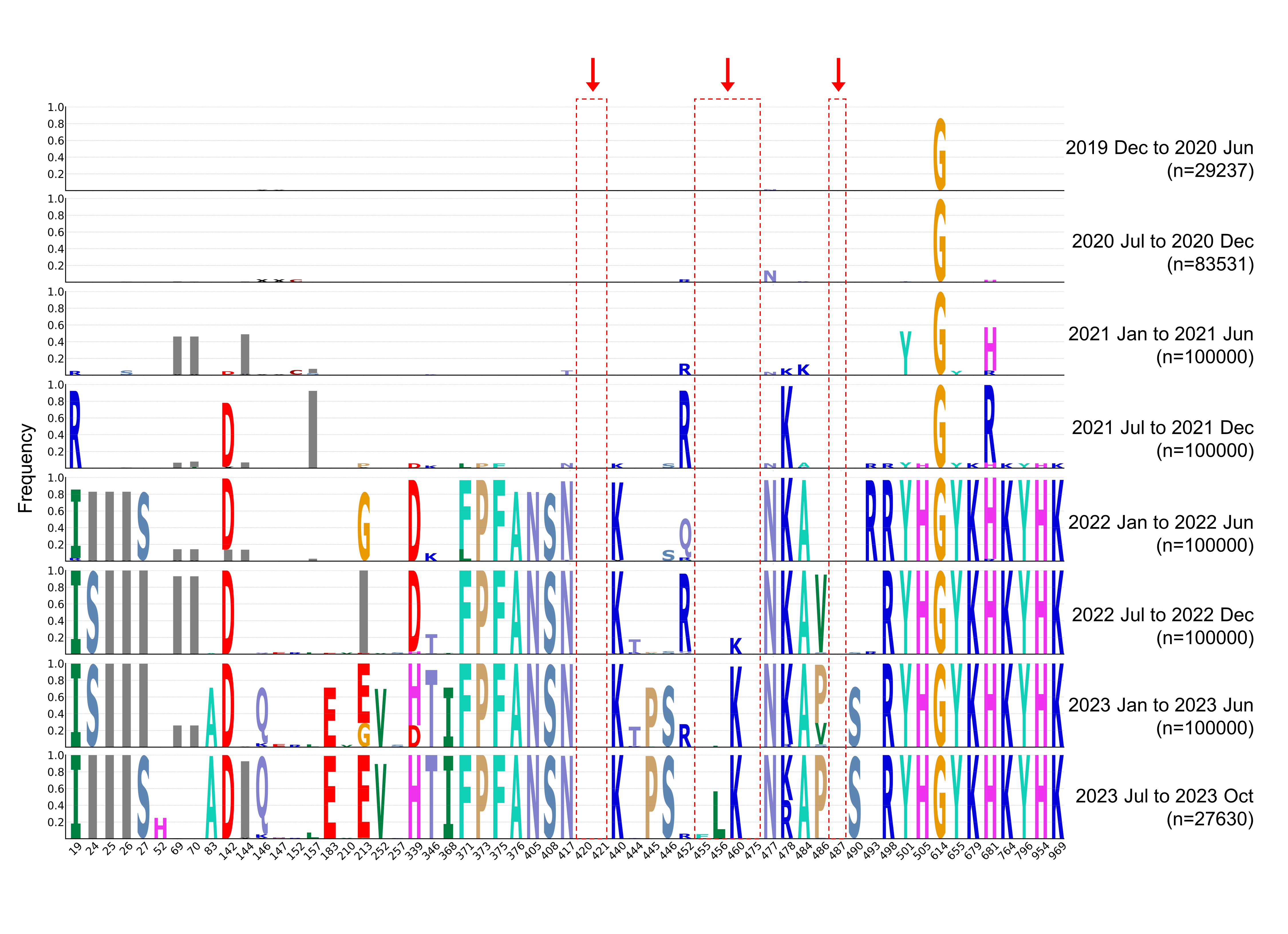

Supplement: S4 Fig — (TIF) [file ppat.1011856.s004.tif]

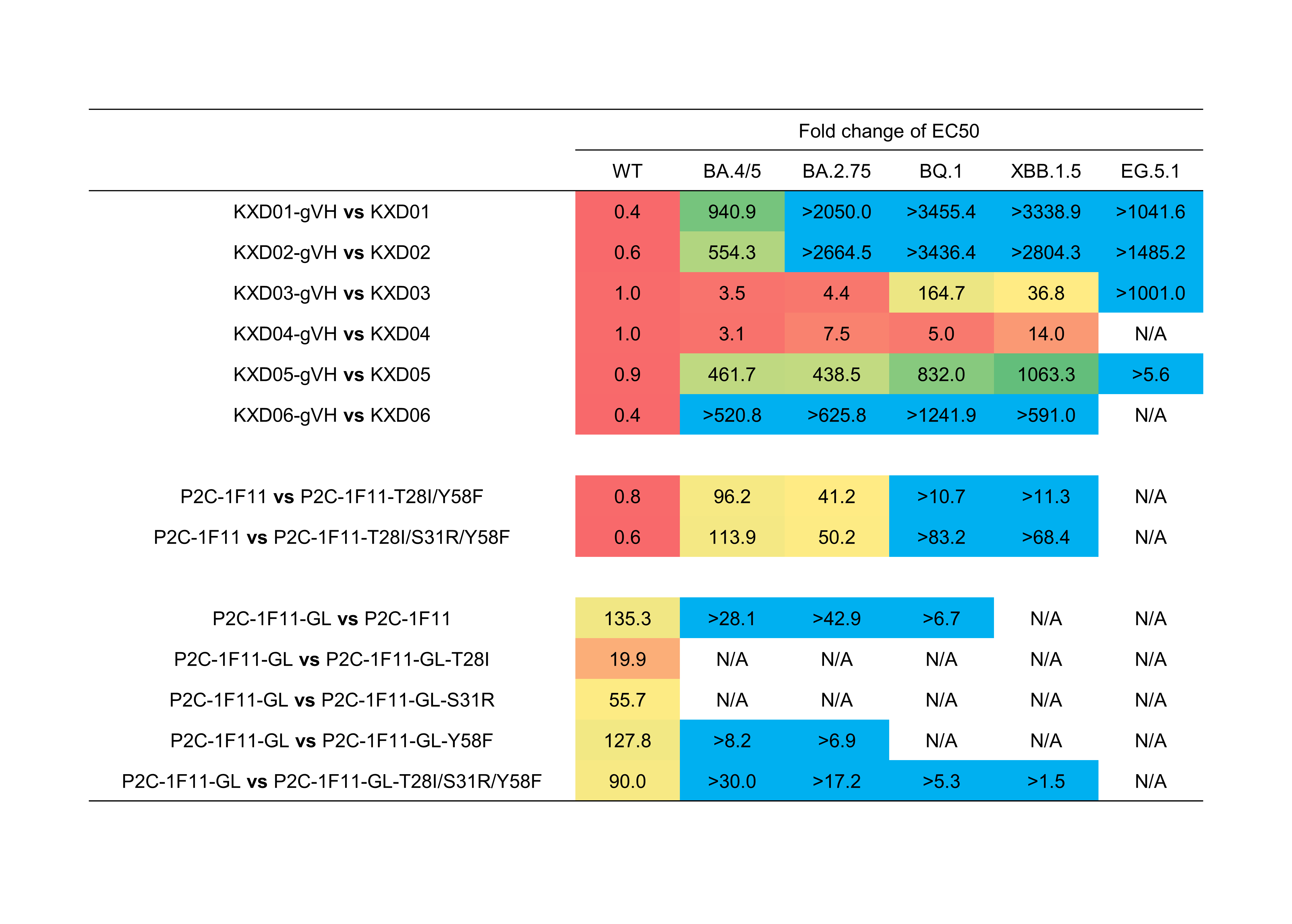

Supplement: S5 Fig — EC50s against RBDs were measured by ELISA with starting concentration of 10 μg/ml. For antibodies with poor binding activity to RBDs, EC50s were taken as >10 μg/ml. To calculate the fold change of EC50, higher values were used as numerator and lower values were used as denominator. If both EC50s were >10 μg/ml, then “N/A” is recorded, indicating not applicable. If the numerator is >10 μg/ml, then “>” is used in the fold change. The values in the table were color-coded. (TIF) [file ppat.1011856.s005.tif]
