# Supplementary material for: Breakthrough infection elicits hypermutated IGHV3-53/3-66 public antibodies with broad and potent neutralizing activity against SARS-CoV-2 variants including the emerging EG.5 lineages
Source: PLoS Pathog. 2023 Dec 4;19(12):e1011856. doi: 10.1371/journal.ppat.1011856 (PMC10721163; doi:10.1371/journal.ppat.1011856)
Supplement: Attachment — Submitted filename: Response letter-20231018.docx [file ppat.1011856.s008.docx]

Dear editors and reviewers,

Thank you so much for reviewing our manuscript. After revising our manuscript thoroughly according to your constructive comments, I would like to resubmit our revised manuscript. Below are our responses to your comments and the changes we have made in our revised manuscript. Looking forward to hearing from you.

Sincerely yours,

Teng Zuo

Thanks for submitting the paper. We have received two expert reviews that suggest some important revisions. Please submit a revised version that responds to all of these comments, including especially discussing new mutations such as at site 456 in the RBD that are becoming common in current SARS-CoV-2 variants.

**Response**: We thank the editors and reviewers for their constructive comments. We believe that our paper has been strengthened significantly by addressing them. Regarding to the editors’ suggestion about discussing new mutations such as at site 456 in the RBD that are becoming common in current SARS-CoV-2 variants, we have measured the neutralizing activity of plasma and mAbs against EG.5, EG.5.1, FL.1.5, FL.1.5.1 and EG.5.1+L455F (Figure 1C, D and Figure 3). We have also added the following paragraph to discuss these results in the discussion (Line 321-328):

**Regarding to the surveillance of SARS-CoV-2, emerging variants like EG.5, EG.5.1, FL.1.5 and FL.1.5.1 have F456L mutation, which falls into the escape maps of KXD01-06. Although these variants show similar resistance to plasma neutralization as XBB and XBB.1, they partially escape the neutralization of KXD01-03 and completely escape the neutralization of KXD04-06. Moreover, addition of L455F can further increases the neutralization resistance of EG.5.1 to KXD01-03. Consistent with studies published recently(9, 10), these results demonstrate the evolution potential of SARS-CoV-2 and highlight the need to monitor current variants with those mutations.**

Reviewer's Responses to Questions

**Part I – Summary**

Please use this section to discuss strengths/weaknesses of study, novelty/significance, general execution and scholarship.

Reviewer #1: IGHV3-53/3-66 genes encode a major public class of neutralizing antibodies targeting the receptor binding site of the SARS-CoV-2 spike protein. Although a vast majority of IGHV3-53/3-66 antibodies are evaded by SARS-CoV-2 variants, exceptions have been discovered - some IGHV3-53/3-66 antibodies exhibited broad reactivity and neutralizing activity against the variants. In this manuscript, the authors identified six IGHV3-53/3-66 antibodies that can neutralize a broad range of variants from WT to XBB.1.16. However, while breakthrough infections eliciting IGHV3-53/3-66 antibodies with great breadth and potency have been widely reported and discussed, the authors should highlight the new observations and added knowledge from this study.

**Response**: We thank Reviewer #1 for suggesting us to highlight the new observations and added knowledge from this study.

The first notable finding of this study is that the antibody response in these two convalescents is dominated by IGHV3-53/3-66 antibodies with broad neutralizing capacity. Although similar antibodies have been widely reported and discussed, it is not reported that they are dominant in convalescents or vaccinees. To highlight this point, we have revised the title of our manuscript to “**Breakthrough infection elicits dominant IGHV3-53/3-66 public antibodies with broad and potent neutralizing activity against SARS-CoV-2 variants including the emerging EG.5 lineages**” (Line 1-3). We have also revised the abstract by adding “**the dominance of broadly neutralizing IGHV3-53/3-66 public antibodies in these two convalescents provides rationale for novel vaccines based on this class of antibodies**” (Line 27-29). Based on this observation, we primarily discussed the development of broadly neutralizing IGHV3-53/3-66 antibodies in our original version. In this revised version, we have highlighted this point again in discussion by adding “**The identification of these two convalescents and KXD01-06 suggest that broadly neutralizing IGHV3-53/3-66 antibodies can be highly dominant in antibody response against SARS-CoV-2, although the underlying mechanism remains to be explored**” (Line 265-268). Overall, we believe that this finding is an important support for vaccine development aiming at eliciting this class of bnAbs.

Another new finding of this study is the identification of KXD03, which uses a long CDRH3 (21 amino acids by IMGT numbering) and exhibits broad neutralizing capacity. Although broadly neutralizing IGHV3-53/3-66 antibodies have been widely reported, few of them use long CDRH3 (more than 15 amino acids). Therefore, KXD03 represents a unique type of IGHV3-53/3-66 antibodies. To highlight this point, we have added the following discussion (Line 314-320). “**On the other hand, although IGHV3-53/3-66 antibodies with broadly neutralizing activity have been widely reported and characterized (8, 19-23, 28, 41-43), few of them are from RBD class 2. Here we show that KXD03 is able to neutralize all tested SARS-CoV-2 viruses and is even more potent to variants with L455F than KXD01 and KXD02. Taken together, these observations suggest that KXD03 represents a unique type of IGHV3-53/3-66 antibodies with long CDRH3, which will be characterized structurally in the future.”**

Reviewer #2: The authors identified a panel of broadly neutralizing antibodies against multiple SARS-CoV-2 variants including XBB lineages, named KXD01-06, which belong to the IGHV3-53/3-66 public antibody family and target the ACE2-binding sites on the virus RBD. Although these antibodies exhibit attractive neutralization breadth in the manuscript’s characterization, some additional validation experiments are necessary and some concerns should be addressed before my recommendation for publication.

**Response**: We thank Reviewer #2 for providing the following constructive comments. As shown below, we have addressed all those comments.

**Part II – Major Issues: Key Experiments Required for Acceptance**

Please use this section to detail the key new experiments or modifications of existing experiments that should be absolutely required to validate study conclusions.

Generally, there should be no more than 3 such required experiments or major modifications for a "Major Revision" recommendation. If more than 3 experiments are necessary to validate the study conclusions, then you are encouraged to recommend "Reject".

Reviewer #1: 1. Deep mutational scanning experiments here have demonstrated that some mutations on residues 455, 456, and 460 can lead to KXD01-06 antibody escape (Figure 4). Variants EG.5.1 and FL.1.5.1 are rapidly rising and contain mutations at these positions. Can the authors investigate the potential impact of these emerging variants on these antibodies? Based on these considerations, relevant sections of the manuscript may need to be modified, for example:

Line 24: “conserved sites on RBD including D420, Y421, L455, F456, A475 and N487”

Lines 48-49: “the emerging BQ and XBB sublineages, display most striking neutralization evasion” – the new variants may be even more resistant to antibody neutralization (DOI: 10.1101/2023.08.21.553968).

Lines 187-189: “KXD01-06 target highly conserved epitopes and they can be escaped by future variants with mutations on residues including D420, Y421, L455, F456, N460, A475 and N487”

Line 173: “mutations are only found in SARS-CoV-1 on L455, F456”

**Response**: We thank Reviewer #1 for providing these helpful suggestions. We have measured the binding activity of KXD01-06 and other mAbs to EG.5.1 RBD, which shows that KXD01-03 maintain binding to EG.5.1 RBD whereas KXD04-06 substantially lose binding to EG.5.1 RBD (Figure 3). Moreover, we have measured the neutralizing activity of those mAbs against EG.5, EG.5.1, FL.1.5, FL.1.5.1 and EG.5.1+L455F, which shows that these variants partially or completely escape the neutralization of KXD01-06. We have revised result section accordingly (Line 145-161).

We have revised “conserved sites on RBD including D420, Y421, L455, F456, A475 and N487” to “**KXD01-06 can be escaped by current and prospective variants with mutations on D420, Y421, L455, F456, N460, A475 and N487**” (Line 24-25).

We have revised “the emerging BQ and XBB sublineages, display most striking neutralization evasion” to “**the emerging XBB and EG.5 sublineages, display most striking neutralization evasion**” (Line 50).

We have revised “KXD01-06 target highly conserved epitopes and they can be escaped by future variants with mutations on residues including D420, Y421, L455, F456, N460, A475 and N487” to “**KXD01-06 can be escaped by future variants with mutations on residues including D420, Y421, L455, F456, N460, A475 and N487**” (Line 199-201).

We have revised “mutations are only found in SARS-CoV-1 on L455, F456” to “**only SARS-CoV-1 shows variation on A475**” (Line 182-183).

In addition, we have revised relevant sections throughout the manuscript.

2. Many previous studies have identified IGHV3-53/3-66 bnAbs (some also neutralize XBB), and have extensively discussed potential reasons for their breadth. e.g. DOIs 10.1186/s12929-023-00955-x,10.1038/s41421-022-00449-4,10.1101/2022.01.03.474825, 10.1126/sciimmunol.abo3425. It is important to show new findings of this study.

**Response**: We agree that many previous studies have identified IGHV3-53/3-66 bnAbs and have extensively discussed potential reasons for their breadth. As discussed above regarding to Reviewer #1’s summary, we have some new findings in this study. On one hand, we show that IGVH3-53/3-66 bnAbs are dominant in these two convalescents with the other mAbs isolated from their PBMCs are either moderately neutralizing or non-neutralizing. We believe that this finding is an important support for vaccine development aiming at eliciting this class of bnAbs. On the other hand, we have identified KXD03 with long CDRH3 and broadly neutralizing capacity, which is rare in IGHV3-53/3-66 antibodies. Overall, we believe that this study can extend our understanding about IGHV3-53/3-66 antibodies to some degree.

In addition, we acknowledge that structural characterization is critical to understand the molecular mechanism for broad neutralization and neutralization escape, as exemplified by studies mentioned above and other published studies. Therefore, we have planned to determine the structure of KXD01-06, particularly KXD03, in complex with RBD or spike, which will provide detailed insights into their neutralization breadth and show more new findings.

3. A major claim of this study was that the common somatic mutations VH T28I, S31R and Y58F, when introduced to an IGHV3-53/3-66 antibody, significantly enhanced its affinity and neutralization. A prior study already reported a similar finding, where similar mutations were demonstrated to confer binding and breadth to this class of antibodies (DOI: 10.1101/2022.01.03.474825) but not cited or discussed here. Additionally, it is interesting why P2C-1F11, adding the three mutations, still did not neutralize XBB.

**Response**: We thank Reviewer #1 for pointing this out. We have cited the study mentioned above (Line 231, Line 239, Reference 28). In that study, they introduced individual or combined mutations to the germline of CAB-17. To compare with that study, we have also introduced T28I, S31R and Y58F to the germline of P2C-1F11 in our revised version (Figure 5F, Figure S5). We find that those mutations substantially increase the binding of P2C-1F11 germline to WT RBD while have limited or little effects on the binding of P2C-1F11 germline to BA.4/5, BA.2.75, BQ.1, XBB.1.5 and EG.5.1 RBDs. The results of P2C-1F11 and P2C-1F11 germline suggest that more mutations beyond the common mutations such as T28I, S31R and Y58F are required to confer P2C-1F11 germline high affinity to variant RBDs. Therefore, our major point is that the extent of somatic hypermutation is critical for the neutralization breadth of IGHV3-53/3-66 public antibodies. We have revised relevant sections accordingly (Line 230-238).

Regarding to the poor neutralization of XBB by P2C-1F11 with additional three mutations, although additional mutations substantially increase the binding activity of P2C-1F11 to BQ.1 and XBB.1.5 RBDs, the binding is still not strong enough compared with WT, BA.4/5 and BA.2.75 RBDs (Figure 5E). Therefore, it is likely that the poor neutralization of P2C-1F11 with addition mutations against BQ.1 and XBB lineages to is due to low binding affinity. In our neutralization assay, 10 μg/ml is the starting concentration for dilution. From the raw data shown below, we can observe some neutralization against BQ.1, XBB.1.5 and XBB.1.16 at 10 μg/ml.

|  | BQ.1 | | | | | |
| --- | --- | --- | --- | --- | --- | --- |
| μg/ml | P2C-1F11 | | P2C-1F11-T28I/Y58F | | P2C-1F11-T28I/S31R/Y58F | |
| 0.005 | -56 | -14 | 0 | 1 | 17 | 19 |
| 0.014 | -36 | -59 | -8 | 16 | 13 | 18 |
| 0.041 | -38 | -24 | -14 | -10 | 17 | 27 |
| 0.123 | -45 | -28 | 19 | 5 | 11 | 19 |
| 0.370 | -64 | -19 | 1 | 16 | 17 | 18 |
| 1.111 | -48 | -26 | 5 | 17 | 29 | 46 |
| 3.333 | -60 | -12 | 21 | 27 | 36 | 45 |
| 10 | -41 | -5 | 22 | 41 | 60 | 54 |
|  | | | | | | |
|  | XBB | | | | | |
| μg/ml | P2C-1F11 | | P2C-1F11-T28I/Y58F | | P2C-1F11-T28I/S31R/Y58F | |
| 0.005 | 2 | -4 | 38 | 14 | -13 | -22 |
| 0.014 | -40 | 9 | 5 | 13 | -26 | -63 |
| 0.041 | -15 | 21 | 14 | 39 | 5 | -33 |
| 0.123 | -19 | 5 | 19 | 22 | -6 | -11 |
| 0.370 | 4 | 6 | 6 | 21 | -28 | -26 |
| 1.111 | -14 | -8 | 8 | 17 | -4 | 5 |
| 3.333 | -52 | 7 | 6 | -25 | -9 | -13 |
| 10 | -19 | 1 | 14 | 2 | 5 | 19 |
|  | | | | | | |
|  | XBB.1 | | | | | |
| μg/ml | P2C-1F11 | | P2C-1F11-T28I/Y58F | | P2C-1F11-T28I/S31R/Y58F | |
| 0.005 | 15 | -9 | 7 | 11 | -1 | -21 |
| 0.014 | 11 | 20 | 13 | 14 | 4 | -22 |
| 0.041 | 15 | 8 | 13 | -1 | 17 | -21 |
| 0.123 | 19 | -6 | 0 | -1 | -1 | -20 |
| 0.370 | 37 | 8 | 15 | 7 | 12 | -31 |
| 1.111 | 18 | 24 | -45 | 25 | 18 | 4 |
| 3.333 | -1 | -16 | 1 | -28 | 3 | -30 |
| 10 | 16 | -7 | 14 | -8 | 27 | 20 |
|  | | | | | | |
|  | XBB.1.5 | | | | | |
| μg/ml | P2C-1F11 | | P2C-1F11-T28I/Y58F | | P2C-1F11-T28I/S31R/Y58F | |
| 0.005 | 26 | -19 | -6 | -3 | -10 | -3 |
| 0.014 | -1 | -13 | 16 | 18 | -7 | -13 |
| 0.041 | 2 | -34 | -8 | 21 | -6 | -22 |
| 0.123 | 16 | -19 | 16 | 13 | 1 | -8 |
| 0.370 | -27 | -23 | 32 | -6 | 8 | 10 |
| 1.111 | -18 | -33 | 13 | 6 | -2 | 2 |
| 3.333 | -34 | -16 | -4 | 3 | 12 | 18 |
| 10 | -45 | -12 | 49 | 29 | 61 | 52 |
|  | | | | | | |
|  | XBB.1.16 | | | | | |
| μg/ml | P2C-1F11 | | P2C-1F11-T28I/Y58F | | P2C-1F11-T28I/S31R/Y58F | |
| 0.005 | -17 | 19 | 23 | 37 | 34 | 2 |
| 0.014 | 13 | 20 | 31 | 49 | 29 | 3 |
| 0.041 | -10 | 2 | 30 | 56 | 32 | -1 |
| 0.123 | -8 | 37 | 40 | 55 | 32 | -8 |
| 0.370 | 13 | 29 | 31 | 35 | 29 | -6 |
| 1.111 | 0 | 28 | 45 | 53 | 36 | 16 |
| 3.333 | 24 | 21 | 53 | 53 | 59 | 6 |
| 10 | -2 | 27 | 58 | 59 | 61 | 56 |

To further test whether P2C-1F11 with addition mutations are able to neutralize BQ.1 and XBB lineages, we have repeated the neutralization with 50 μg/ml as the starting concentration and observed neutralization at low level (Figure 5G). We have revised relevant section accordingly (Line 241-242).

Reviewer #2: Major points

1. F456L is a mutation convergently observed in multiple XBB*+486P lineages, such as XBB.1.5.10, EG.5, and FL.1.5. As F456 is exactly on the epitope recognized by the identified IGHV3-53/3-66 neutralizing antibodies, it is crucial to test the activity of KXD01-06 against these XBB variants with F456L, at least using pseudovirus.

**Response**: We thank Reviewer #2 for providing this suggestion. In the revised version, we have measured the neutralizing activity of those mAbs against EG.5, EG.5.1, FL.1.5, FL.1.5.1 and EG.5.1+L455F, which shows that these variants partially or completely escape the neutralization of KXD01-06. We have revised our manuscript accordingly (Line 151-160).

2. Line 175. Considering the evolution and circulation of SARS-CoV-2 VOCs, it is better to sample more recently uploaded sequences, which is much more reasonable, given that previous sequences are not likely to circulate again due to their weak capability of escaping neutralizing antibodies. If you use recent sequences for the analysis, you should find that nearly all sequences carry N460K and F456L is a frequent mutation.

**Response**: We thank Reviewer #2 for pointing this out. We have collected sequences of SARS-CoV-2 spike ranging from 2019 December to 2023 October and analyzed their mutation with WT spike as reference (Figure S4). Just as Reviewer #2 mentioned, N460K appeared in the second half of 2022 and reached 100% quickly. In the past several months, F456L has become a common mutation among the circulating strains along with the emergence of L455F. We have revised our manuscript accordingly (Line 185-192).

3. SHM of IGHV3-53/3-66 is analyzed in the study. However, only the common mutations in published IGHV3-53/3-66 antibodies are summarized in Figure 5C. Can you identify some specific SHMs that exhibit enrichment in the broadly neutralizing antibodies but not the escaped antibodies?

**Response**: We thank Reviewer #2 for raising this interesting question. To figure this out, we collected sequences of 32 broadly neutralizing IGHV3-53/3-66 (BD-bnAbs) from a study performed by Cao Yunlong and colleagues (Figure 5B). By combining KXD01-06 with BD-bnAbs, we have summarized the SHM profile of broadly neutralizing IGHV3-53/3-66 antibodies (Figure 5C top). As a control, we have also summarized the SHM profile of published 1208 IGHV3-53/3-66 antibodies (Figure 5C middle). By comparing the SHM profiles, we find that mutations accumulated by KXD01-06 and BD-bnAbs, such as Y58F, F27I, F27L, T28I, S31R, S35T, S35N and V50L, are also highly frequent among the other IGHV3-53/3-66 antibodies. Therefore, we have not identified any specific SHMs that exhibit enrichment in the broadly neutralizing antibodies but not the escaped antibodies. We have revised our manuscript accordingly (Line 214-220)

4. It is mentioned that Sanger sequencing is used to identify escape mutations in the DMS experiments. However, because the frequencies of yeast cells carrying different variants could be quite different in the library, it is usually necessary to sequence both the mAb-negative yeasts and the pre-antibody-selection yeasts for normalization. This step is not mentioned in the methods. Can you give a detailed methods for the analysis the DMS datasets and the illustration of Figure 4B? The heights of “K” and “R” seem too high in Figure 4B and there may be some artifacts.

**Response**: We thank Reviewer #2 for pointing this out. It is true that the DMS experiments performed by us is not exactly same as published studies.

First, Sanger sequencing is used to identify escape mutations as we realized that Sanger sequencing was more feasible for us after we tried deep sequencing like other groups. In addition, we have shown that Sanger sequencing is also an efficient way to identify escape mutations in our previous publications (doi: 10.1084/jem.20171022.; doi: 10.1038/ncomms9855.;), which can be exemplified by the following figure.

From doi: 10.1084/jem.20171022.

Second, as Reviewer #2 mentioned, pre-antibody-selection yeasts are not sequenced for normalization in the original manuscript. To address this concern, we have sequenced those yeasts in our revised version, which shows that the distribution of mutations is rather random (Figure S3A). Regarding to the analysis the DMS datasets and the illustration of Figure 4B, we acknowledge that normalization is not performed in this process. Therefore, we use frequency for the Y axis instead of escape fraction or escape score. Below is how we analyze the sequences and illustrate the results:

1. The sequences are aligned with reference and mutations are identified as exemplified by the following graph.

2. The frequency of mutation on each position is analyzed and then bar graphs are generated accordingly as exemplified by the following graph.

3. According to the mutation frequency, positions with high mutation frequency are picked out and mutation profiles on these positions are illustrated by sequence-logo graphs as exemplified by the following graph.

Third, we performed an additional round of sorting compared with others. The reason is that most yeasts maintain binding activity with KXD01-06 after negative-selection (Figure 4A and Figure S3B). Moreover, we have also noticed that negative selection enriches yeasts losing binding to ACE2 as well. Therefore, the purpose of the third round of sorting is to further remove mutants maintaining binding to antibodies or losing binding to ACE2. It appears that our selection is more stringent, and thus some mutations are highly enriched as shown by sequence logo graphs. To confirm this explanation, we have sequenced the yeasts after second round of sorting by KXD01. As shown below, the mutations of yeasts after second sorting are more diverse compared with mutations of yeasts after third round of sorting (Figure 4B) and the heights of “K” and “R” are much lower.

We have revised our manuscript accordingly (Line 169-171).

**Part III – Minor Issues: Editorial and Data Presentation Modifications**

Please use this section for editorial suggestions as well as relatively minor modifications of existing data that would enhance clarity.

Reviewer #1: 1. Lines 209-212: “common mutations including T28I, S31R and Y58F … dramatically increase the binding activity of P2C-1F11 to variant RBDs while not to WT RBD”. This observation contrasts with previous findings where Y58F increased binding of this class of antibodies to WT RBD by 10-1000 fold (DOIs: 10.1038/s41467-021-24123-7, 10.1101/2022.01.03.474825). The authors may want to discuss this.

**Response**: We thank Reviewer #1 for pointing this out. This point is relevant with the third major point of Reviewer #1. In our study, these mutations are introduced to P2C-1F11, which has high affinity to WT RBD but lacks common mutations such as Y58F. In previous studies which show that Y58F increased binding of antibody to WT RBD by 10-1000, those antibodies have low affinity to WT RBD before they acquire Y58F. This should be the reason why we have not observed increased binding of P2C-1F11 with additional Y58F mutation to WT RBD.

To further confirm this, we have introduced T28I, S31R and Y58F to the germline version of P2C-1F11 (P2C-1F11-GL) individually or in combination (Figure 5F, Figure S5). In this setting, we find that Y58F substantially increase the binding of P2C-1F11-GL to WT RBD by 127.8 folds, which is consistent with previous findings. Therefore, for affinity-matured IGHV3-53/3-66 antibodies like P2C-1F11, additional Y58F may not increase their binding to WT-RBD. We have revised our manuscript accordingly (Line 230-238)

2. What is the antibody sequence numbering system used in this study? Kabat, IMGT or Chothia? For example, in line 122 “short CDRH3 ranging from 10 to 12 amino acids or long CDRH3 of 21 amino acids”. Different numbering systems can result in varying definitions of CDR lengths. Y58F is used throughout the manuscript but “Y57F” is shown in line 201.

**Response**: We thank Reviewer #1 for pointing this out. IMGT numbering system is used in this study. Y57F should be Y58F. We have revised our manuscript accordingly (Line 126, Line 217).

3. Line 267: “IGHV3-35” should be replaced by “IGHV3-53”

**Response**: We thank Reviewer #1 for pointing this out. This has been corrected in the revised version (Line 296)

4. Line 273: “(ref)”

**Response**: We thank Reviewer #1 for pointing this out. This has been removed in the revised version (Line 302)

5. Line 294: “KCD” should be replaced by “KXD”?

**Response**: We thank Reviewer #1 for pointing this out. This has been corrected in the revised version (Line 336)

Reviewer #2: Minor points

1. Line 72. It will be much clearer for the readers to specify the RBD mutations in the VOCs that are responsible for the escape of IGHV3-53/3-66 antibodies (mainly K417N and N460K).

**Response**: We thank Reviewer #2 for providing this suggestion. In our revised version, we have added mutations including N417T/K, N460K, E484K/A and F486V/S (Line 74-75)

2. Line 129. The names of the two prototype NAbs of Evusheld should be COV2-2196/2130 instead of CoV-2196/2130. This should be corrected throughout the manuscript to avoid confusion.

**Response**: We thank Reviewer #2 for pointing this out. We have corrected them throughout the manuscript in revised version.

3. Line 201. The authors seem to use an uncommon numbering of antibody chains. For example, a frequent SHM on IGHV3-53/3-66 is previously referred to as Y58F, but here Y57F is used (https://www.nature.com/articles/s41467-021-24123-7; https://www.science.org/doi/full/10.1126/science.abd2321). Please check the numbering.

**Response**: We thank Reviewer #2 for pointing this out. IMGT numbering is used in this manuscript. Y57F should be Y58F. We have corrected this in our revised version (Line 126, Line 217).
