# Supplementary material for: Breakthrough infection elicits hypermutated IGHV3-53/3-66 public antibodies with broad and potent neutralizing activity against SARS-CoV-2 variants including the emerging EG.5 lineages
Source: PLoS Pathog. 2023 Dec 4;19(12):e1011856. doi: 10.1371/journal.ppat.1011856 (PMC10721163; doi:10.1371/journal.ppat.1011856)
Supplement: Attachment — Submitted filename: Response letter-20231121.docx [file ppat.1011856.s009.docx]

Dear editors and reviewers,

Thank you so much for reviewing our revised manuscript and providing additional comments to refine it. After addressing those constructive comments, I would like to submit a new version of our manuscript. Below are our responses to your comments and the changes we have made in our revised manuscript. Looking forward to hearing from you.

Sincerely yours,

Teng Zuo

Thanks for submitting your revised manuscript. The reviewers are generally supportive. They note (and I agree) that these antibodies may soon be partially escaped by new mutations at sites like 455, 456, and 475 that are present in some of the newest variants. Nonetheless, this remains an interesting study. I just ask that you address the minor comments provided by the reviewers and provide a tracked changes manuscript in the resubmission. If you do that, I should be able to editorially review it and accept it provided you address the minor comments.

Response: We thank the editors and reviewers for their support. As shown below, we have addressed those constructive comments.

Reviewer's Responses to Questions

Part I - Summary

Please use this section to discuss strengths/weaknesses of study, novelty/significance, general execution and scholarship.

Reviewer #1: The authors have addressed most of my comments. I still have two questions.

1. In the revised manuscript, the authors demonstrated that the neutralization by IGHV3-53/3-66 antibodies was reduced or evaded by variants containing F456L and/or L455F (Figure 3). Nonetheless, their plasma exhibited a comparable level of neutralization across variants with and without F456L/L455F mutations (Figure 1 and Response “… these variants show similar resistance to plasma neutralization as XBB and XBB.1 …”). This suggests that antibodies other than IGHV3-53/3-66 could contribute significantly to the plasma neutralization. Consequently, the revised emphasis on the “dominance of IGHV3-53/3-66 antibodies” may not be precise.

Response: We thank Reviewer #1 for pointing this out. In our study, the dominance of IGHV3-53/3-66 is highlighted based on the observation that those antibodies are prevalent among 50 spike-specific mAbs isolated from two convalescents. We agree that antibodies other than IGHV3-53/3-66 could contribute significantly to the plasma neutralization. Indeed, we mention that those 50 mAbs may be not enough to represent the antibody response in the two individuals when we discuss the potential limitations of our study (Line 342-344).

To be more precise, we have revised the title “**Breakthrough infection elicits dominant IGHV3-53/3-66 public antibodies with broad and potent neutralizing activity against SARS-CoV-2 variants including the emerging EG.5 lineages**” to “**Breakthrough infection elicits hypermutated IGHV3-53/3-66 public antibodies with broad and potent neutralizing activity against SARS-CoV-2 variants including the emerging EG.5 lineages**” (Line 1-3).

We have revised “**the dominance of broadly neutralizing IGHV3-53/3-66 public antibodies in these two convalescents**” to “**the prevalence of broadly neutralizing IGHV3-53/3-66 public antibodies in these two convalescents**” (Line 27).

We have revised “**these bnAbs are dominant in the two convalescents**” to “**these bnAbs are prevalent in the two convalescents**” (Line 41).

We have revised “**These data suggest that the dominant neutralizing antibodies from these two convalescents belong to previously defined IGHV3-53/3-66 public antibodies**” to “**These data suggest that a large portion of neutralizing antibodies from these two convalescents belong to previously defined IGHV3-53/3-66 public antibodies**” (Line 141).

We have revised “**which once again confirms the dominance of IGHV3-53/3-66 antibodies in neutralizing antibodies against SARS-CoV-2**” to “**which once again confirms the prevalence of IGHV3-53/3-66 antibodies in neutralizing antibodies against SARS-CoV-2**” (Line 262)

In addition, we have revised “**The identification of these two convalescents and KXD01-06 suggest that broadly neutralizing IGHV3-53/3-66 antibodies can be highly dominant in antibody response against SARS-CoV-2, although the underlying mechanism remains to be explored**” to “**The identification of these two convalescents and KXD01-06 suggest that broadly neutralizing IGHV3-53/3-66 antibodies can be highly abundant in antibody response against SARS-CoV-2, although the underlying mechanism remains to be explored**” (Line 280).

2. The authors have included a discussion in the revised manuscript (Lines 314-320) that states:

“On the other hand, although IGHV3-53/3-66 antibodies with broadly neutralizing activity have been widely reported and characterized (8, 19-23, 28, 41-43), few of them are from RBD class 2. Here we show that KXD03 is able to neutralize all tested SARS-CoV-2 viruses and is even more potent to variants with L455F than KXD01 and KXD02. Taken together, these observations suggest that KXD03 represents a unique type of IGHV3-53/3-66 antibodies with long CDRH3, which will be characterized structurally in the future.”

However, IGHV3-53/3-66 antibodies with a long CDR H3 are not novel. For instance, C144 (DOI: 10.1038/s41586-020-2852-1) and COVA2-39 (DOI: 10.1016/j.celrep.2020.108274) have long CDR H3s.

Moreover, while most IGHV3-53/3-66 antibodies with long CDRH3 target class 2 epitope, some bind to the class 1 epitope. For example, the IGHV3-66 antibody BG1-22 has a 19-AA long CDR H3 but is a class 1 antibody (10.1016/j.cell.2021.04.032). In fact, the definition of class 1-4 antibodies is largely based on structural, or at least precise epitope information (10.1038/s41586-020-2852-1). Classifying RBD antibodies without detailed data is challenging.

Response: We thank Reviewer #1 for pointing this out. We agree with Reviewer #1’s points that IGHV3-53/3-66 antibodies with a long CDR H3 are not novel and it is challenging to classify RBD antibodies without structure and epitope information. We want to clarify that we are not taking KXD03 as a RBD class 2 antibody. Moreover, the major point we want to highlight is that broadly neutralizing IGHV3-53/3-66 antibodies generally use short CDRH3, whereas KXD03 is a broadly neutralizing IGHV3-53/3-66 antibody with long CDRH3, which is not widely characterized. To make this point clearer, we have revised the discussion as below.

“**On the other hand, although IGHV3-53/3-66 antibodies with broadly neutralizing activity have been widely reported and characterized (8, 19-23, 28, 41-43), they generally use short CDRH3. Here we show that KXD03, which has a long CDRH3, is able to neutralize all tested SARS-CoV-2 viruses and is even more potent to variants with L455F than KXD01 and KXD02. Taken together, these observations suggest that KXD03 represents a unique type of IGHV3-53/3-66 antibodies with long CDRH3, which will be characterized structurally in the future.**” (Line 329-333)

Reviewer #2: The authors have provided convincing explanation and satisfactory additional experiments and analyses to address my concerns. Although the highlighted mAbs exhibited reduced neutralization activity against latest XBB subvariants with F456L mutation, which may dampen the significance of this study, the analyses and data shown in the study are basically sound and meaningful, helping understand the maturation of IGHV3-53/3-66 public antibodies against SARS-CoV-2 and variants. The long CDR-H3, and the role it played in the neutralization breadth of KXD03 and other IGHV3-53/3-66 mAbs, is also of interest and importance. However, I have the following concerns and suggestions about the modified manuscript.

Response: We thank Reviewer #2 for recognizing our study. As shown below, we have addressed all the concerns and suggestions.

Part II – Major Issues: Key Experiments Required for Acceptance

Please use this section to detail the key new experiments or modifications of existing experiments that should be absolutely required to validate study conclusions.

Generally, there should be no more than 3 such required experiments or major modifications for a "Major Revision" recommendation. If more than 3 experiments are necessary to validate the study conclusions, then you are encouraged to recommend "Reject".

Reviewer #1: (No Response)

Reviewer #2: (No Response)

Part III – Minor Issues: Editorial and Data Presentation Modifications

Please use this section for editorial suggestions as well as relatively minor modifications of existing data that would enhance clarity.

Reviewer #1: (No Response)

Reviewer #2: 1.EG.5, EG.5.1, and FL.1.5 should have exactly the same RBD sequences, but as shown in Figure 3, KXD01-03 exhibit distinct neutralization against them. Specifically, FL.1.5 seems more resistant to the mAbs than EG.5.1. KXD03 exhibits much lower neutralization against EG.5 (0.764 ug/mL) than EG.5.1 (0.097 ug/mL). I recommend repeating the neutralization assays to confirm the results, if the authors did not perform the assays in replicates, or is it possible to explain them?

Response: We thank Reviewer #2 for pointing this out. As noted in figure legend for Figure 3: **All results are representatives of two independent experiments, in which duplicates are performed** (Line 623-625). The IC50s presented in Figure 3 are from one batch of independent experiments. And the IC50s of KXD01-03 against EG.5, EG.5.1, FL.1.5 and FL.1.5.1 from the two batches are compared below.

|  |  | Neutralization (IC50:μg/ml) | | | |
| --- | --- | --- | --- | --- | --- |
|  |  | EG.5 | EG.5.1 | FL.1.5 | FL.1.5.1 |
| Batch 1 | KXD01 | 0.074 | 0.190 | 0.515 | 0.565 |
|  | KXD02 | 0.118 | 0.163 | 0.670 | 0.621 |
|  | KXD03 | 0.764 | 0.097 | 0.427 | 0.533 |
|  | | | | | |
| Batch 2 | KXD01 | 0.026 | 0.175 | 0.450 | 0.633 |
|  | KXD02 | 0.458 | 0.134 | 0.448 | 0.390 |
|  | KXD03 | 0.433 | 0.663 | 0.181 | 0.738 |

Overall, the IC50s from the two batches are close. As pointed out by Reviewer #2, the IC50 of KXD03 against EG.5.1 is 0.097 μg/ml. In the second batch, the IC50 of KXD03 against EG.5.1 is 0.663 μg/ml, which is about 7-fold higher than the value in Batch 1.

To further confirm the above results, we have sequenced the plasmids encoding the spikes of EG.5, EG.5.1, FL.1.5 and FL.1.5.1 again. In addition, neutralization assays have been repeated again by three members in our group.

And the IC50s from 5 batches are compared below.

|  |  | Neutralization (IC50:μg/ml) | | | |
| --- | --- | --- | --- | --- | --- |
|  |  | EG.5 | EG.5.1 | FL.1.5 | FL.1.5.1 |
| Batch 1 | KXD01 | 0.074 | 0.190 | 0.515 | 0.565 |
|  | KXD02 | 0.118 | 0.163 | 0.670 | 0.621 |
|  | KXD03 | 0.764 | 0.097 | 0.427 | 0.533 |
|  | | | | | |
| Batch 2 | KXD01 | 0.026 | 0.175 | 0.450 | 0.633 |
|  | KXD02 | 0.458 | 0.134 | 0.448 | 0.390 |
|  | KXD03 | 0.433 | 0.663 | 0.181 | 0.738 |
|  | | | | | |
| Batch 3 | KXD01 | 0.806 | 0.300 | 0.401 | 0.535 |
|  | KXD02 | 0.242 | 0.031 | 0.070 | 0.111 |
|  | KXD03 | 0.448 | 0.214 | 0.373 | 0.288 |
|  | | | | | |
| Batch 4 | KXD01 | 0.543 | 0.171 | 0.536 | 0.352 |
|  | KXD02 | 0.221 | 0.047 | 0.362 | 0.065 |
|  | KXD03 | 0.403 | 0.115 | 0.425 | 0.227 |
|  | | | | | |
| Batch 5 | KXD01 | 0.573 | 0.505 | 0.526 | 0.345 |
|  | KXD02 | 0.169 | 0.218 | 0.217 | 0.037 |
|  | KXD03 | 0.497 | 0.331 | 0.191 | 0.145 |

Overall, these IC50s are consistent between different batches except the values highlighted with colors. According to this dataset, it seems that EG.5, EG.5.1, FL.1.5 and FL.1.5.1 maintain similar neutralization sensitivity to KXD01-03. We speculate that the differences observed by Reviewer #2 is due to batch variations in our neutralization assays and we apologize for these misleading data. We have combined the data from 5 batches of neutralization assays to calculate the IC50s, which are shown below and have replaced the original values in Figure 3.

|  | Neutralization (IC50:μg/ml) | | | |
| --- | --- | --- | --- | --- |
|  | EG.5 | EG.5.1 | FL.1.5 | FL.1.5.1 |
| KXD01 | 0.349 | 0.253 | 0.514 | 0.507 |
| KXD02 | 0.225 | 0.100 | 0.376 | 0.196 |
| KXD03 | 0.489 | 0.238 | 0.340 | 0.341 |

2.The authors provided the sequencing results of pre-selection DMS library in Fig. S3A at residue level. Please show the frequencies of each mutation in the pre-selection library, in order to check whether the K/R mutants on residues 420, 421, 456, 475 etc. are originally abundant in the library. If so, it will explain why there are exceptional enrichment of K/R in the DMS results of mAbs, which may be artifacts. Is it possible to normalize the results by the frequency of each mutation in the library before selection?

Response: We thank Reviewer #2 for providing this suggestion. We have shown the frequencies of each mutation in the pre-selection library (Figure S3B). Based on Figure S3B, K/R mutations on residues 420, 421, 456, 475 etc. are not found in the pre-selection library. On the other hand, we have only sequenced limited number of yeast clones from the library before selection. As a result, most enriched mutations after selection are not found in the sequences before selection. Therefore, we are not able to normalize the frequency of enriched mutations by the frequency of each mutation in the library before selection.

3.The Spike sequences or mutations of variants involved in this study should be provided as a Supplementary table or figure.

Response: We thank Reviewer #2 for providing this suggestion. We have provided a supplementary table containing the spike sequences. Meanwhile, we have realized that spike sequence of EG.5.1+L455F is equal to spike sequence of HK.3. Therefore, we have replaced EG.5.1+L455F with HK.3 across our revised manuscript. We have also replaced XBB.1.5.70 with HK.3 in Figure 4C.
